# A scoping review of type 2 diabetes mellitus in Pakistan investigating the status of glycemic control, awareness, treatment adherence, complications and cost

**DOI:** 10.3389/fendo.2024.1441591

**Published:** 2024-11-22

**Authors:** Hira Taimur, Ishtiaq Ahmad, Hamza Khan, Yoshihisa Shirayama, Miyoko Okamoto, Myo Nyein Aung, Sameera Shabbir, Motoyuki Yuasa

**Affiliations:** ^1^ Department of Global Health Research, Graduate School of Medicine, Juntendo University, Tokyo, Japan; ^2^ Advanced Research Institute for Health Sciences, Juntendo University, Tokyo, Japan; ^3^ Faculty of International Liberal Arts, Juntendo University, Tokyo, Japan; ^4^ Central Campus, International Higher School of Medicine, Bishkek, Kyrgyzstan

**Keywords:** T2DM, glycemic control, awareness, medication adherence, complications, cost, Pakistan

## Abstract

**Background:**

The high prevalence of Type 2 diabetes mellitus(T2DM) in Pakistan is a challenge to the existing healthcare system. This is the first comprehensive review of the status of glycemic control, diabetes knowledge, treatment adherence, complications and financial burden faced by the diabetic patient population of the country.

**Methods:**

We searched PubMed, Web of Science and Scopus for studies on diabetes control, knowledge, treatment adherence, prevalence of complications and cost in Pakistan published in English from 2000 to 2024. We hand-searched Google Scholar for additional papers and included a total of 45 studies in our review.

**Results:**

The review shows that poor glycemic control prevails among diabetic patients ranging from 44.7% to 86.4% along with half of the patients have poor diabetes knowledge (46.0% -70.0%). Treatment adherence level in diabetic patients varies widely in different studies, frequently reported complications are retinopathy (14.5%-43.0%), nephropathy (14.0%-31.0%) and neuropathy (10.8%-59.6%); and the disease poses a great deal of economic burden.

**Conclusion:**

Most of the studies were observational. Glycemic control and knowledge among individuals with T2DM in Pakistan are inadequate, leading to a high prevalence of complications that impose significant health and economic burdens. Further longitudinal studies generating evidence of lifestyle modifications as primary and secondary prevention strategies against diabetes in the Pakistani population can form a strong foundation for awareness campaigns and policy revisions.

## 1 Introduction

In recent times, Type 2 diabetes (T2DM) has emerged as one of the most common non-communicable diseases in high as well as in low and middle-income countries (1). According to WHO, T2DM was the largest cause of mortality in 2019 claiming 1.5 million lives (2). As per the International Diabetes Federation (IDF) Atlas 2021, 10.5% of the adult global population (20-79 years) has diabetes. Furthermore, by 2045, 783 million adults will be diabetic (one in eight adults) as per IDF projections (3). The dynamics of this disease are changing. It is estimated that globally T2DM accounts for 12% of health expenditure in 2010 at $376 billion and is expected to reach $490 billion in 2030 (4). T2DM is one of the fastest-rising public health concern in Asian countries mainly due to lifestyle changes associated with economic transitions, globalization and urbanization (5).

**Figure 1:**
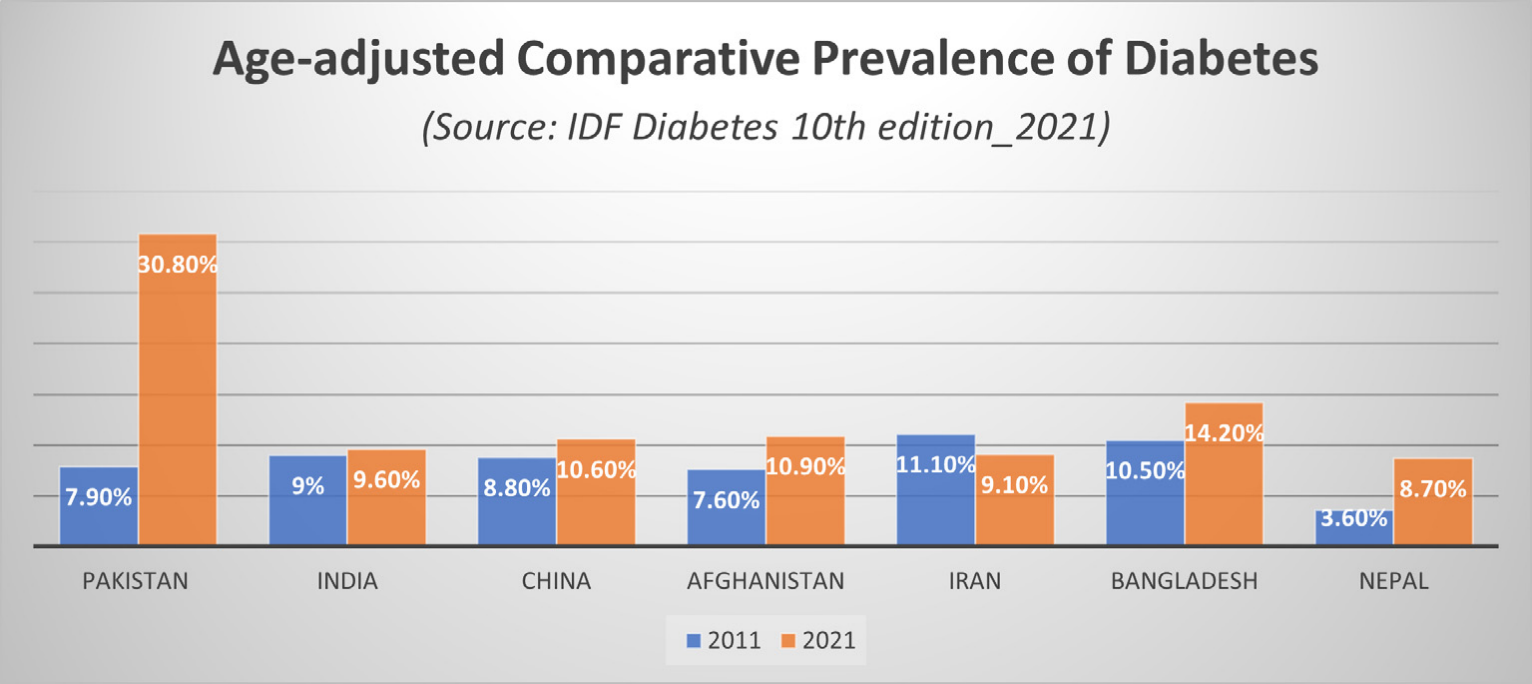
Age-adjusted comparative prevalence of diabetes. Source: IDF Diabetes Atlas 10^th^ edition 2021.

Pakistan is a low-middle-income country located in South Asia. According to the second National Diabetic Survey of Pakistan NDSP (2016-2017), the weighted prevalence of diabetes was 26.3%. (28.3% in urban areas and 25.3% in rural areas), while the prevalence of pre-diabetes was 14.4% (6). Pakistan is the 5^th^ most populous country in the world. The most recent IDF Atlas estimated that 33 million people are living with diabetes in Pakistan, which constitutes 3^rd^ largest diabetes population globally (7). An additional 11 million adults have impaired glucose tolerance, while 8.9 million with diabetes remain undiagnosed making it a major public health concern in the country (8). The age-adjusted comparative prevalence of diabetes among the 20 to 79 years age group in Pakistan has been compared to regional countries in **Figure 1** (data obtained from IDF Diabetes Atlas 10^th^ edition 2021 (3)). The high prevalence can be attributed to the poor state of non-communicable disease risk factors in the country such as unhealthy diet, physical inactivity, increasing trends of tobacco consumption, overweight and obesity (9, 10).

An important aspect of diabetes in resource-limited settings like Pakistan is its economic impact. High expenses can quickly drain household and national resources thereby deterring poverty reduction initiatives. The real burden of this disease is due to chronic complications which lead to increased morbidity and mortality. Thus, in addition to the primary prevention strategies, secondary prevention of diabetic complications in individuals who have already developed the disease is equally essential in reducing the health and economic burden of T2DM. A cornerstone for improving diabetes outcomes and proper management is patient education about the disease (11). Lower mortality and positive health outcomes in people living with T2DM have also been found to be associated with good treatment adherence (12–14). On the other hand, poor glycemic control is linearly correlated with microvascular complications of diabetes (15–17). With the current prevalence statistics, every fourth adult in Pakistan is diabetic. We, therefore, consider it important to examine the situation of people living with this disease. Our objective is to collate data on the status of glycemic control among diabetic patients, diabetic knowledge, compliance to treatment, the prevalence of complications and cost; with the aim to help tailor secondary prevention strategies according to the needs of the diabetic population of the country.

## 2 Methods

### 2.1 Study design

The goal of a scoping review is to map the literature on a broad research theme and to identify the gaps. It is a preferred study design when there are uncertainties and/or deficiencies in the volume of literature. We conducted this scoping review based on the York methodology outlined by Arksey and O’Malley (18) with the aim to capture all literature regardless of study design and quality. As suggested by the framework, we followed the five stages: (1) identifying the research question (2) identifying relevant studies (3) selecting appropriate studies (4) data charting and (5) collating summarizing and reporting results (**Supplementary File 1**). The conduct of this review was consistent with the Preferred Reporting Items for Systematic Reviews and Meta-analysis (PRISMA-ScR) Extension for Scoping Reviews (19).

### 2.2 Eligibility criteria

Inclusion criteria were: (1) articles published from 2000 to 2024 (2) articles published in the English language (3) articles that addressed one of the five main areas of this review (prevalence of uncontrolled diabetes, complications, cost, medication adherence and awareness) and (4) articles for which full text was obtained for this review.

Exclusion criteria were: (1) studies that do not fulfill the above criteria (2) review papers reporting the results of other studies (3) studies conducted among the Pakistani diaspora residing abroad.

### 2.3 Search strategy

We used relevant search terms with appropriate Boolean operators and filters to comprehensively search studies published in the English language between 2000 to 2024 on different databases (PubMed, Web of Science, Scopus and Google Scholar). (**Supplementary File 2**). Necessary adjustments were made in the search term depending on the database used.

### 2.4 Selection of studies

**Figure 2:**
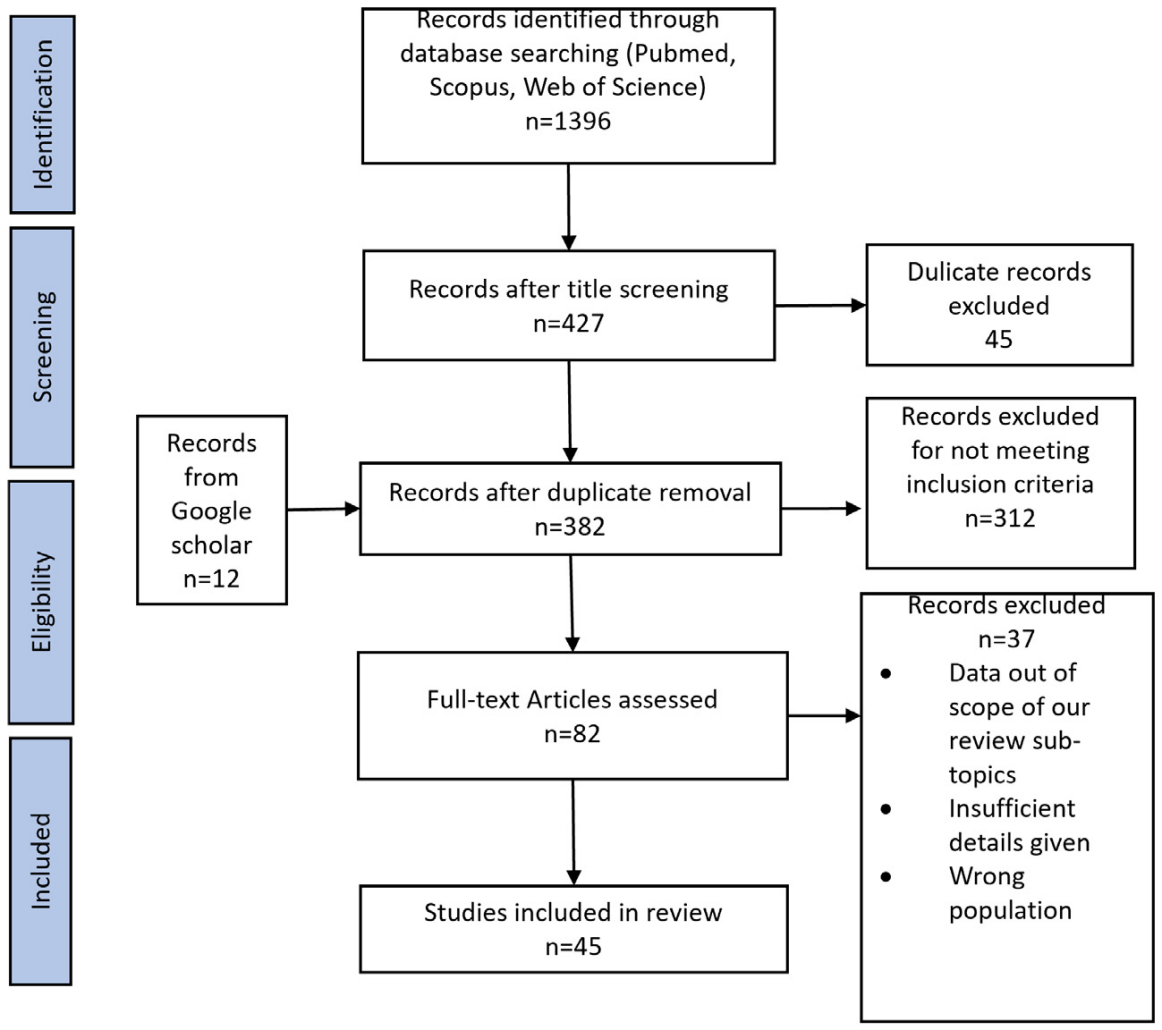
PRISMA flow chart.

The search identified a total of 1396 studies from the abovementioned databases. Initially, the titles of the identified studies were screened independently for relevance. Titles deemed relevant by any evaluator were retained for abstract screening. Subsequently, the evaluators examined the abstracts of retained studies to identify those deemed worthy of full article screening. Discussions were performed in case of any disagreement until an agreement was reached regarding article inclusion. Lastly, full text was examined for final inclusion. Two reviewers (HT and IA) extracted the data and an impartial third reviewer provided advice regarding article selection. Step-wise article selection process is shown in the PRISMA flow chart in **Figure 2**.

### 2.5 Data items and charting process

For all studies reviewed, data were extracted and charted regarding sample size, study population, study setting and instrument/criteria used for results. The extracted data from all sources was transferred to Endnote by three reviewers (HT, HK and SS). Collated data was rechecked by the third reviewer (IA) and matched with original sources. A separate excel sheet was generated for data about each theme from where the relevant data was summarized and charted in main manuscript. A full list of articles according to sub-themes is given in **Supplementary File 3**. Other than charting, we conducted a narrative descriptive synthesis of determinants and factors associated with the results from each theme. Assessment of risk bias and appraisal of quality of included sources was not done. The characteristics of the included studies are given in **Table 1**.

**Table 1:** Characteristics of included studies.

ID no.	Principal Author/Publication Year	Sample size	Study site/ Setting	Study population and age range	Study Design	Sampling Technique
1	Atif M et al., 2019 (20)	400	Lahore/ Public hospitals	Elderly Pwd >65 years old, diagnosed at least 6 months prior	Cross-sectional descriptive	Convenience
2	Khowaja MA et al., 2023 (21)	451	Karachi/ Private hospital	Pwd, all ages	Cross-sectional	Purposive
3	Hai AA et al., 2019 (22)	288	Karachi/ Public hospital	Pwd >=18 years	Cross-sectional	Consecutive
4	Farooque R et al., 2020 (23)	329	Karachi/ Public hospital	Pwd >14 years	Prospective cross-sectional	Not mentioned
5	Bukhsh A et al., 2018 (24)	218	Islamabad, Lahore/ Public hospital and private clinics	Pwd >30 years diagnosed 1 year prior	Cross-sectional	Not mentioned
6	Sayeed KA et al., 2020 (25)	317	Karachi/ Hospital based (sector not mentioned)	Pwd>=45years, diagnosed at least 6 months prior	Cross-sectional	Not mentioned
7	Arshad R et al., 2023 (26)	766	Lahore/ Private hospital	Pre-diagnosed Pwd, all ages	Prospective cross-sectional	Consecutive
8	Fawwad A et al., 2018 (27)	4633	Karachi/ Public hospital	Pre-diagnosed Pwd, all ages	Retrospective longitudinal follow-up	Not mentioned
9	Jawad N et al., 2017 (28)	332	Karachi/ Private hospital	Pre-diagnosed Pwd, all ages	Cross-sectional analytical	Not mentioned
10	Akhter J et al., 2017 (29)	876	Karachi/ Registry based	Pre-diagnosed Pwd, all ages	Cross-sectional	Random
11	Athar MH et al., 2020 (30)	190	Rawalpindi/ Public hospital	Pwd diagnosed at least 1 year prior, all ages	Cross-sectional analytical	Purposive
12	Noor A et al., 2021 (31)	188	Peshawar/ Public hospital	Pre-diagnose Pwd, >=18 years	Prospective observational	Not mentioned
13	Khan AU et al., 2013 (32)	650	Rawalpindi/ Public hospital	pre-diagnosed Pwd, >=35 years	Cross-sectional descriptive	Not mentioned
14	Aziz A et al., 2021 (33)	94	Karachi/ Private hospital	Pre-diagnosed pwd, hospitalized for any reason >=18 years	Retrospective observational	Consecutive
15	Basit A et al., 2004 (34)	2199	Karachi/ Public hospital	Pwd, all ages	Cross-sectional analytical	Not mentioned
16	Siddiqui F J et al., 2015 (35)	452	Karachi/ Community based specialized care center	Pwd diagnosed atleast 3 months prior, >=25 years	Cross-sectional survey	Consecutive
17	Shera AS et al., 2004 (36)	500	Karachi/ Public hospital	Pre-diagnosed, pwd >=25 years	Cross-sectional survey	Random sampling
18	Sultana R et al., 2021 (37)	300	Nawabshah/ Private clinics	Pwd diagnosed atleast 6 months prior, >=34 years	Prospective cross-sectional	Simple random
19	Ramzan B et al., 2022 (38)	151	Multan/ Private hospital	Pwd, all ages	Cross-sectional	Simple random
20	Iqbal Q et al., 2017 (39)	300	Quetta/ Public and private hospitals	Pre-diagnosed Pwd, >=18 years	Cross-sectional descriptive	Not mentioned
21	Khan A et al., 2021 (40)	212	Rawalpindi/ Public hospital	Pre-diagnosd Pwd, all ages	Cross-sectional analytical	Consecutive
22	Shams N et al., 2016 (41)	163	Islamabad/ Private hospital	Pwd,>=65 years	Cross-sectional descriptive	Consecutive
23	Uthman M et al., 2015 (42)	250	Lahore/ Public hospital	Pwd, 40-70 years	Cross-sectional descriptive	Not mentioned
24	Anwar I etal, 2022 (43)	1180	District Faisalabad/ Family medicine clinics	Pwd, >=14 years	Cross-sectional	Not mentioned
25	Kumar A etal, 2021 (44)	135	Larkana/ Public hospital	Pwd, 35-60 years	Cross-sectional descriptive	Not mentioned
26	Butt MD et al., 2023 (45)	388	Islamabad, Rawalpindi Quetta/ Public and private hospitals	Pre-diagnosed Pwd, >=18 years	Cross-sectional	Not mentioned
27	Nazir S et al., 2016 (46)	392	Sargodha/ Public hopital	Pre-diagnosed Pwd, all ages	Cross-sectional	Not mentioned
28	Sarwar H et al., 2014 (47)	140	Hyderabad/ Public hospitals	Pwd diagnosed 1 year prior, 18-80 years	Quantitative descriptive	Random
29	Beg BM et al., 2018 (48)	432	Lahore/ Public hospitals	Pre-diagnosed Pwd, >=18 years	Cross-sectional	Convenient
30	Shams N et al., 2020 (49)	200	Rawalpindi/ Private hospital	Pwd, Group 1 <65 years, Group 2 <=65 years	Cross-sectional descriptive	Convenient
31	Iqbal SP et al., 2023 (50)	151	Karachi/ Private hospital	Pwd, all ages	Cross-sectional	Convenient
32	Abro M et al., 2019 (51)	28,601	Karachi/ Private hospital	Pwd, all ages	Retrospective cross-sectional	Not mentioned
33	Zia A et al., 2016 (52)	678	Islamabad/ Public hospital	Pwd, all ages	Cross-sectional	Not mentioned
34	Asghar S et al., 2023 (53)	160	Rahim Yar Khan / Public hospital	Pwd with metabolic syndrome, >=20 years	Prospective cohort	Not mentioned
35	Uddin F et al., 2019 (54)	891	Multicenter (20 cities) Registry-based	Pwd, >=18 years	Cross-sectional (registry based)	Simple random
36	Sharif S et al., 2019 (55)	100	Lahore/ Public hospital	Pwd, >=35 years	Cross-sectional	Purposive
37	Akhtar MS et al., 2011 (56)	210	District Jhang/ Public hospitals and private clinics	Type 1 and 2 diabetics, 5-85 years	Cross-sectional	Not mentioned
38	Wahab S et al., 2008 (57)	130	Karachi/ Public hospital	Pwd, all ages	Cross-sectional	Not mentioned
39	Khan KA etal,2015 (58)	200	Rawalpindi/ Public hospital	Pwd, 40-70 years	Cross-sectional descriptive	Consecutive
40	Hussain S et al., 2013 (59)	299	Bahawalpur/ Public hospital	Pwd>=30 years	Cross-sectional descriptive	Purposive
41	Tahir MM et al., 2021 (60)	200	Lahore/ Private hospital	Newly diagnosed Pwd, 30-60 years	Cross-sectional descriptive	Consecutive
42	Butt MD et al., 2022 (45)	1839	Multicenter/ Outpatient clinics throughout the country	Pre-diagnosed Pwd, >=18 years	Cross-sectional survey	Cluster
43	Khwaja LA et al., 2007 (61)	345	Karachi/ Public and private clinics	Pre-diagnosed Pwd, 20-60 years	Prevalence-based cost of illness study	Systematic random
44	Datta BK et al., 2019 (62)	24,238 households	All over Pakistan/ Community-based study	General population	Cross-sectional survey	Not mentioned
45	Hussein M et al., 2014 (63)	885	Karachi/ Private hospitals	Pwd, 20-60 years	Prevalence-based cost of illness study	Random

Pwd, Patients with Type 2 diabetes.

## 3 Results

### 3.1 Uncontrolled diabetes and poor glycemic control

Among different cross-sectional studies conducted in the country, poor glycemic control ranged from 44.7% to 86.4% (cutoff value of Hba1c varied a little among studies) as shown in **Table 2**. Factors associated with poor glycemic control mentioned by Saeed et al., (64) are compromised self-care, poor adherence to medication, lack of social support and low literacy level (25). Arshad R et al., (26) documented in their study that only 9% of female diabetic patients had normal BMI, which explained a higher prevalence of poor glycemic control in women as compared to men in that study (26). The compliance of checking Hba1c was also found to be inadequate (58.5%) in diabetic patients by Aziz et al., (33). Anwer et al. (65) in his study on 1180 diabetic patients from urban and rural areas of the Faisalabad district documented that 40% of diabetic patients never had their Hba1c checked (43).

**Table 2:** Glycemic control in diabetic patients.

Reference	Percent prevalence of poor glycemic control	Criteria for poor glycemic control (%)
Atif M et al., 2019 (20)	67.8%	Hba1c>=7
Khowaja MA et al., 2023 (21)	63.4%	Hba1c>=7
Hai AA et al., 2019 (22)	81.6%	Hba1c>=7
Farooque R et al., 2020 (23)	70.8%	Hba1c>7
Bukhsh A et al., 2018 (24)	83.0%	Hba1c>=7
Sayeed KA et al., 2020 (25)	60.6%	Hba1c>=7
Arshad R et al., 2023 (26)	86.4%	Hba1c>=7
Fawwad A et al., 2018 (27)	85.9%	Hba1c>7
Jawad N et al., 2017 (28)	72.7%	Hba1c>=7
Akhter J et al., 2017 (29)	52.9%	Hba1c>8
Athar MH et al., 2020 (30)	44.7%	Hba1c>8
Noor A et al., 2021 (31)	83.5%	Hba1c>=7
Khan AU et al., 2013 (32)	58.0%	Hba1c>8
Shera AS et al., 2004 (36)	46.2%	Hba1c>7
Aziz A et al., 2021 (33)	73.4%	Hba1c>=7
Basit A et al., 2004 (34)	81.3%	Hba1c>7
Siddiqui FJ et al., 2015 (35)	74.0%	Hba1c>7

### 3.2 Diabetes awareness

The knowledge and awareness about the disease in the diabetic population is generally poor as shown in **Table 3**. Anwar I et al., 2022, a survey conducted in rural and urban areas of Faisalabad district on 1180 diabetic patients found better knowledge of diabetic complications in women compared to men (43). Shams et al. (66) has documented illiteracy, poverty, poor glycemic control, poor dietary control and use of alternate modes of therapy as factors associated with poor diabetes knowledge in elderly diabetics (41). Uthman M et al., (42) found in their KAP (Knowledge, Attitude, Practice) study that good diabetes knowledge exists in 63.0% of respondents, however good practice towards diabetes was found in 7.0% only (42). Kumar A et al., (44) found inadequate knowledge of the symptoms and self-management of hypoglycemia in 42.5% of T2DM (44).

**Table 3:** Diabetes knowledge among people living with diabetes.

Reference	Percent Knowledge	Instruments used to measure diabetes knowledge
Sultana R et al., 2021 (37)	Good knowledge 9.3%, Moderate knowledge 21.7%, Poor knowledge 69%	Diabetes Knowledge Questionnaire (DKQ)
Ramzan B et al., 2022 (38)	Good knowledge 3.9%, Average knowledge 15.2%, Poor knowledge 79.4%	Michigan Diabetes Knowledge Test (MDKT)
Iqbal Q et al., 2017 (39)	Good knowledge 2.3%, Moderate knowledge 27.6%, Poor knowledge 70.0%	Michigan Diabetes Knowledge Test (MDKT)
Khowaja MA et al., 2023 (21)	Adequate knowledge 96.7%, Inadequate knowledge 3.3%	Diabetes Knowledge Questionnaire (DKQ-24)
Khan A et al., 2021 (40)	Good knowledge 40.6%, Moderate-poor knowledge 59.4%	Diabetes Knowledge Questionnaire
Shams N et al., 2016 (41)	Good knowledge 24.5%, Moderate knowledge 22.7%, Poor knowledge 52.8%	Michigan Diabetes Knowledge Questionnaire (DKQ-24)
Uthman M et al., 2015 (42)	Good knowledge 63.0%, Moderate knowledge 39.0%, Poor knowledge 46.0%	KAP (Knowledge, Attitude, Practice) Questionnaire

### 3.3 Diabetes treatment adherence

Standard treatments for controlling glucose levels include oral hypoglycemics and insulin. In Pakistan, Metformin has the highest (51.3%) prescription rate followed by insulin/analogues (34.6%) (45). Butt MD et al., (45) documented in their recent multicenter cross-sectional study that the majority (26%) of patients with T2DM received triple therapy while 34.7% received monotherapy (45). Adherence to treatment according to our selected papers varies according to the study site as shown in **Table 4**.

**Table 4:** Treatment adherence in diabetic patients.

Reference	Adherence percentage	Instrument used to measure adherence
Butt MD et al., 2023 (45)	Good Adherence 59.6%, Moderate adherence 35.7%, Poor adherence 4.7%	Drug Attitude Inventory (DAI-10)
Khowaja MA et al., 2023 (21)	Adherence 82.3%, Non-adherence 17.7%	Brief Medication Questionnaire
Nazir S et al., 2016 (46)	Moderate to High adherence 28.1%, Low adherence 71.9%	Morisky Medication Adherence Scale (MMAS-U)
Ramzan B et al., 2022 (38)	Good adherence 59.6%, Moderate adherence 37.1%, Poor adherence 3.3%	Drug Attitude Inventory (DAI-10)
Sarwar H et al., 2014 (47)	Good adherence 14.3%, Moderate adherence 45.7%, Low adherence 40.0%	Morisky scale
Beg BM et al., 2018 (48)	High adherence 15.7%, Medium adherence 43.1%, Low adherence 41.2%	Morisky Medication Adherence Scale (MMAS-8)
Iqbal Q et al., 2017 (39)	Good Adherence 55.6%, Moderate adherence 37.0%, Poor adherence 7.3%	Drug Attitude Inventory (DAI-10)
Shams N et al., 2020 (49)	<65 years: High 10%, Moderate 32.0%, Low 58.0% >=65 years High 3%, Moderate 24.0%, Low 73.0%	Morisky Medication Adherence Scale (MMAS-8)

A survey conducted in Karachi by Iqbal SP et al., (39) documented that patients cited side effects of medicines as the most common factor related to non-compliance followed by forgetfulness, complex regime and financial constraints (50). Polypharmacy, combined anti-diabetic regimes, visual morbidity and physical dependence were the factors associated with poor adherence in geriatric diabetic patients according to Shams N et al., (49). Beg BM et al., (48) found in their study conducted in Lahore that the majority of patients who were non-adherent had low literacy levels (48).

### 3.4 Prevalence of diabetic complications

Micro and macrovascular complications of diabetes increase the disease burden on patients as well as the health system. A study conducted by Zia A et al., (52), in the capital, documented the percent prevalence of diabetic macrovascular complications as ischemic heart disease 28.2%, stroke 8.5%, peripheral vascular disease 5.4% (52). Uddin et al. (67) in their national, multicenter observational study conducted across 20 cities in Pakistan documented micro and macrovascular complication prevalence to be 68.6% and 9.0% respectively with commonly observed macrovascular complications being angina (5.2%), myocardial infarction (3.3%), peripheral arterial disease (2.2%) and stroke (2.0%) (54). Basit et al., in 2004 documented hypertension as the most common macrovascular complication in Karachi (50.4%) followed by coronary artery disease (15.1%) (34). The prevalence of microvascular complications is given in **Table 5**.

**Table 5:** Microvascular complications of diabetes.

Reference	Percent prevalence	Clinical Setting of Study	Identification of complication
Abro M et al., 2019 (51)	Retinopathy 15.8% Nephropathy 31.0% Neuropathy 48.7%	Baqai Institute of Diabetology and Endocrinology, Karachi	Objective examination
Zia A et al., 2016 (52)	Retinopathy 0.8% Nephropathy 1.0% Neuropathy 7.3%	PIMS and KRL hospitals, Islamabad	From medical records
Asghar S et al., 2023 (53)	Retinopathy 24.9% Nephropathy 16.8% Neuropathy 10.8%	Sheikh Zayed Hospital, Rahim Yar Khan	Objective examination
Uddin F et al., 2019 (54)	Retinopathy 15.9% Nephropathy 24.4% Neuropathy 59.6%	Multicentral across 20 cities	From Medical records
Sharif S et al., 2019 (55)	Retinopathy 22.0% Nephropathy 14.0% Neuropathy 16.0%	Jinnah Hospital, Lahore	From medical records
Akhtar MS et al., 2011 (56)	Retinopathy 30.0% Nephropathy 29.5% Neuropathy 38.1%	Various clinics from District Jhang	Objective examination
Basit A et al., 2004 (34)	Retinopathy 15.9% Nephropathy 28.4% Neuropathy 36.6%	Baqai Institute of Diabetology, Karachi	From medical records
Shera AS et al., 2004 (36)	Retinopathy 43.0% Nephropathy 20.2% Neuropathy 39.6%	Clinic of Diabetic Association of Pakistan, Karachi	Objective examination
Wahab S et al., 2008 (57)	Retinopathy 15.0%	Dow University of Health Sciences, Karachi	Objective examination
Khan KA etal,2015 (58)	Retinopathy 14.5%	Military Hospital, Rawalpindi	Objective examinations
Hussain S et al., 2013 (59)	Retinopathy 23.9%	Bahawal Victoria Hospital, Bahawalpur	Objective examination
Tahir MM et al., 2021 (60)	Peripheral Neuropathy 21.0%	Fatima memorial Hospital, Lahore	Objective examination

### 3.5 Cost and financial burden

A recent multicenter study conducted by Butt MD et al., (68) documented the total annual cost of diabetes in Pakistan to be $740 per person, out of which direct cost was $646.7, while indirect cost was $93.65 (68). Although at the lower end of the general global trend (direct cost: $242 to $11,917, indirect cost: $45 to $16,914 (1)), this is a substantially large expenditure relative to the average income in Pakistan. Hussain M et al., (63) in his survey in Karachi sampling 885 diabetic patients documented that the largest cost is accounted for by medication followed by physician consultation, with none of the participants indicating any coverage by insurance or employer (63). Diabetes management accounts for 62.0% of the per capita income in Pakistan (with a per capita income of $1193.73 as per World Bank data, (69)) (68). A recent nationally representative survey in Pakistan found that catastrophic health expenditure is 6.7 percentage points higher for households consuming blood pressure and diabetes medication (62). A prevalence-based cost of illness study conducted in Karachi by Khawaja LA et al., 2007 documented that 18.0% of total family income is spent on diabetes care (61). Based on the prevalence of T2DM in Pakistan, as reported by International Diabetes Foundation, Butt MD et al., (68) computed the total burden of diabetes management to be $24.42 billion which constitutes 1.6% of Pakistan’s GDP (World Bank data, 2021) (68).

## 4 Discussion

The rising diabetes burden is a major concern in Pakistan. We found in our review that the majority of the diabetic population in Pakistan has poor glycemic control. Many studies pointed out a relatively higher prevalence of poor glycemic control among people belonging to low socioeconomic classes and with lower education levels; which is consistent with overall health inequity prevailing in the country. People belonging to lower socioeconomic classes tend to adhere less to medication and lifestyle modifications eventually leading to further deterioration of glycemic control (70). With the exception of one study (Arshad et al., (26)), glycemic control did not differ significantly between men and women. However, previous reviews on the Pakistani population have documented a higher prevalence of obesity in Pakistani women as compared to men (71, 72), which can be one of the contributing factors towards poor control of diabetes. Women in Pakistani culture are deprived of outdoor leisure exercise facilities especially in rural areas (26). Other contributing factors such as differences in lifestyle factors and genetic predispositions should be explored in future studies. Our findings are consistent with other studies which found poor diabetes control in most low-middle-income countries (73, 74). Additionally, we found poor compliance of diabetes patients with Hba1c testing.

The poor control of diabetes can be explained by other findings of this study such as poor awareness and unsatisfactory medication adherence among patients with diabetes. We included studies that used validated instruments to measure medication adherence and diabetes knowledge. The studies assessing diabetes knowledge had consistent results of poor diabetes knowledge in the majority of patients except the study conducted by Khowaja et al., (21). It was a hospital-based study conducted in the endocrinology clinics of Aga Khan Hospital, Karachi (21). The inconsistency may be due to the fact that this hospital has patient education program in place whereby brochures with frequently asked questions about diabetes and other diseases are provided to the patients and caregivers (75). Also, a hospital-based study conducted in specialized clinics may give better results in terms of disease awareness since better-aware patients are more likely to visit these specialized clinics. It is important to impart relevant disease knowledge to diabetic patients to improve awareness in order to achieve long term glycemic control and prevent complications. Evidence-based and culturally tailored free of cost education programmes should be developed and made available to people living with diabetes especially those with poor access to health facilities.

Studies assessing medication adherence showed substantial variance in results with particularly lower adherence in geriatric patients with diabetes. Inadequate medication adherence is found in the literature to be a major challenge, especially in developing countries (76, 77). However, we found that very few studies (48–50) explored the determinants of poor adherence in the Pakistani population. The main determinants that we found in our review are fear of side effects, illiteracy, complex medication regimens and high cost of medicines. Current scheme of universal health coverage in Pakistan does not cover outpatient consultation or medicine cost. Thus, high cost of medicines coupled with low awareness hinders medication adherence. Patient education about doses and side-effects of medicines and improving doctor-patient relationship can also be beneficial. Further studies in this area can help guide the researchers and decision-makers in selecting appropriate interventions and tailoring them to target the specific determinants of poor medication adherence in the country.

One of the well-known outcomes of poor diabetes control is microvascular complications such as retinopathy, neuropathy and nephropathy. The current review found a high prevalence of these complications in Pakistan. Excluding statistics from one study (Zia A et al., (52)), the prevalence of diabetic retinopathy was 14.5%-43.0%, nephropathy 14.0%-31.0% and neuropathy 10.8%-59.6%. The study by Zia A. et al., (52) gives very small percent prevalence of microvascular complications as compared to the rest of the studies. The author attributes this inconsistency to differences in socioeconomic and lifestyle factors since the study was conducted in two tertiary-care hospitals in the capital city of Islamabad (52). Diabetes-related complications contribute to high financial burdens and low quality of life. Awareness regarding complications in diabetes patients was found to be better in 41-65 years age group and women (43). Comparable figures of microvascular complication prevalence in regional countries are found in the literature (78–80). Precise understanding of the rural-urban variations and trends in diabetes-related complications remains a gap in Pakistan for which we recommend further studies in this domain.

Overall, the current financial burden associated with this disease in the country is very high. Our review shows that high medicine price is a significant contributor to the management cost of diabetes in Pakistan. A systematic review found diabetes medicine price as the biggest burden of the cost of this disease in the neighbouring country of India as well (81). Ensuring access and affordability of diabetes medication is, therefore, crucial to achieve better disease control in developing countries. Devising new healthcare policies and introducing new generic medicines should also be considered.

To the best of our knowledge, this review is the first to collate articles related to the above-mentioned themes in the diabetic patient population of Pakistan. We used robust methods in screening, extracting and reviewing the data. A timely review of the literature and diverse inclusion criteria are additional strengths of this study. The interpretation of the findings of this review has to be done in light of certain limitations. First, there was limited evidence to provide valid estimates at regional and national levels and according to patient characteristics such as gender, age etc. Second, most of the included studies are cross-sectional surveys. So, it is not possible to identify cause and effect. Third, since the study was intended to be a scoping review and not systematic, we did not do quality assessment or risk of bias for included studies. However, we included only those studies which used validated instruments to measure outcomes.

The government has taken many steps to control the burden of diabetes. Important ones include the recent opening of the Diabetes Registry of Pakistan (DROP) (82); the National Association of Diabetes Educators of Pakistan (NADEP), established in 2010 (83); and the Diabetes Prevention Program (DPP) by the Diabetes Association of Pakistan (84). To increase awareness, massive education campaign is needed. We recommend the use of digital platforms which is a cost-effective population-based approach. In Pakistan, the health system in rural and urban slum communities is successfully strengthened by Lady health workers at household and community levels. However, currently, they have a focused scope of maternal, child and reproductive health (85). We recommend widening their scope to include diabetes prevention and control. This will help produce an equitable improvement of diabetes control in the country. Therefore, training of lady health workers and primary health care professionals about the diagnosis and monitoring of diabetes along with diabetes prevention through lifestyle behaviors modification would be a timely investment to reduce the growing burden of diabetes. Collaborative approaches with health organizations in local and national communities will enhance the effect and reach of already existing initiatives. Ongoing evaluation of current interventions and programs is also critical. Meanwhile, it is important to secure universal access to diagnosis, treatment and monitoring of diabetes mellitus to the public.

## 5 Conclusion

The findings of this review highlight the dismal status of diabetes control and inadequate level of diabetes knowledge in Pakistan. The prevalence of diabetic complications is high and the disease poses a significant financial burden on diabetic patients as well as the health system. A well-organized education campaign is needed to raise awareness in this regard. We have included our recommendations for the advancement of diabetes research and prevention strategies in Pakistan. We hope that current strategies and future directions can successfully cater to the rising diabetes menace in the country.
