# Supplementary material for: A scoping review of type 2 diabetes mellitus in Pakistan investigating the status of glycemic control, awareness, treatment adherence, complications and cost
Source: Front Endocrinol (Lausanne). 2024 Nov 22;15:1441591. doi: 10.3389/fendo.2024.1441591 (PMC11621625; doi:10.3389/fendo.2024.1441591)
Supplement: Supplementary file 1 [file DataSheet1.docx]

Supplementary Material

**Supplementary File 1**: Framework stages followed

**Stage 1** Identifying the research question

Identifying the research question

**Stage 2** Identifying relevant studies

Identifying relevant studies

**Stage 3** Study selection

Study selection

**Stage 4** Charting the data

Charting the data

**Stage 5** Collating, summarizing and reporting the results

Collating, summarizing and reporting the results

*We skipped the optional 6^th^ stage of Consultation*

**Supplementary File 2**: Results of literature research with three databases

| **Keyword Search** | **Date of Search** | **Search Engine and number of publications retrieved** | **Articles Selected** |
| --- | --- | --- | --- |
| Diabetes[Mesh Major Topic]AND Pakistan[All Feilds] AND(glycemic[All Feilds])AND((control[All Feilds])OR(poor[All Feilds])OR(uncontrolled[All Feilds])) | 2024/1/6 | PubMed 20  Web of science 221  Scopus 50 | 17 |
| Diabetes[Mesh Major Topic]AND Pakistan[All Feilds]AND((awareness[All Fields])OR(knowledge[All Feilds])) | 2024/1/3 | PubMed 13  Web of science 210  Scopus 215 | 8 |
| Diabetes[Mesh Major Topic]AND Pakistan[All Feilds]AND((treatment[All Feilds])OR(medication[All Feilds]))AND((adherence[All Feilds])OR(compliance[All Fields])) | 2024/1/3 | PubMed 7  Web of science 71  Scopus 59 | 6 |
| Diabetes[Mesh Major Topic]AND Pakistan[All Fields]AND((complications[All Fields])OR(microvascular[All Fields])OR(retinopathy[All Fields])OR(neuropathy[All Fields])OR(nephropathy[All Fields])) | 2024/1/2 | PubMed 19  Web of science 223  Scopus 131 | 10 |
| Diabetes[Mesh Major Topic]AND Pakistan[All Fields]AND((cost[All Fields])OR(financial[All Fields])) | 2024/1/2 | PubMed 10  Web of science 129  Scopus 18 | 4 |
| Total |  | 1,396 | 45 |

**Supplementary File 3: List of articles by sub-themes**

**Glycemic control**

1. Sayeed, K.A., et al., *Impact of diabetes-related self-management on glycemic control in type II diabetes mellitus.* Cureus, 2020. **12**(4).
2. Arshad, R., et al., *Effect of obesity, socioeconomic status and gender on glycemic control in diabetic patients.* Rawal Medical Journal, 2023. **48**(3): p. 651-651.
3. Aziz, A. and S.A. Ali, *Compliance of checking HbA1c in a tertiary care hospital of Pakistan.* Pakistan Journal of Medical Sciences, 2021. **37**(1): p. 142.
4. Atif, M., et al., *Extent and Predictors of Poor Glycaemic Control among Elderly Pakistani Patients with Type 2 Diabetes Mellitus: A Multi-Centre Cross-Sectional Study.* Medicina, 2019. **55**(1): p. 21.
5. Khowaja, M.A., et al., *Factors associated with Diabetes Empowerment among patients with type 2 diabetes, at OPD setting, Karachi, Pakistan.* Scientific Reports, 2023. **13**(1): p. 7165.
6. Hai, A.A., et al., *Diabetes self-care activities and their relation with glycemic control in patients presenting to The Indus Hospital, Karachi.* Cureus, 2019. **11**(12).
7. Farooque, R., et al., *The frequency of poor sleep quality in patients with diabetes mellitus and its association with glycemic control.* Cureus, 2020. **12**(11).
8. Bukhsh, A., et al., *Association of diabetes-related self-care activities with glycemic control of patients with type 2 diabetes in Pakistan.* Patient preference and adherence, 2018: p. 2377-2385.
9. Fawwad, A., et al., *Incidence of microvascular complications of type 2 diabetes: A 12 year longitudinal study from Karachi-Pakistan.* Pak J Med Sci, 2018. **34**(5): p. 1058-1063.
10. Jawad, N., et al., *ANTHROPOMETRY AND DIABETIC CONTROL IN PAKISTAN.* INDO AMERICAN JOURNAL OF PHARMACEUTICAL SCIENCES, 2017. **4**(10).
11. Akhter, J., et al., *Patterns, control and complications of diabetes from a hospital based registry established in a low income country.* BMC Endocrine Disorders, 2017. **17**(1): p. 30.
12. Athar, M.H., et al., *Impact of general education status on glycemic control in patients of diabetes mellitus.* Pakistan Armed Forces Medical Journal, 2020. **70**(Suppl-1): p. S26-30.
13. Noor, A., et al., *Relationship of Homocysteine With Gender, Blood Pressure, Body Mass Index, Hemoglobin A1c, and the Duration of Diabetes Mellitus Type 2.* Cureus, 2021. **13**(11): p. e19211.
14. Khan, A.U., et al., *Status of glycemic control in patients of type 2 diabetes mellitus.* Pakistan Armed Forces Medical Journal, 2013. **63**(2): p. 275-278.
15. Basit, A., et al., *Frequency of chronic complications of type 2 diabetes.* J Coll Physicians Surg Pak, 2004. **14**(2): p. 79-83.
16. Siddiqui FJ, Avan BI, Mahmud S, Nanan DJ, Jabbar A, Assam PN. Uncontrolled diabetes mellitus: prevalence and risk factors among people with type 2 diabetes mellitus in an Urban District of Karachi, Pakistan. Diabetes research and clinical practice. 2015;107(1):148-56.
17. Shera AS, Jawad F, Maqsood A, Jamal S, Azfar M, Ahmed U. Prevalence of chronic complications and associated factors in type 2 diabetes. J Pak Med Assoc. 2004;54(2):54-9.

**Diabetes Awareness**

1. Khowaja, M.A., et al., *Factors associated with Diabetes Empowerment among patients with type 2 diabetes, at OPD setting, Karachi, Pakistan.* Scientific Reports, 2023. **13**(1): p. 7165.
2. Shams, N., et al., *Diabetes knowledge in elderly type 2 diabetes mellitus patients and association with glycemic control.* Journal of Liaquat University of Medical & Health Sciences, 2016. **15**(2).
3. Uthman, M., Z. Ullah, and N.U. Shah, *Knowledge, attitude and practice (KAP) survey of type 2 diabetes mellitus.* Age (years), 2015. **40**(50): p. 80.
4. ANEEL KUMAR, Z.A.S., SHAM LAL PRITHIAN, BASHIR AHMED SHAIKH, IMDAD ALI ANSARI, IQRA HYDER, *Knowledge of Hypoglycemic Symptoms and their Self-Management among patients with Type II Diabetes Mellitus.* Pakistan Journal of Health and Medical Sciences, Nov, 2021. **15**(11).
5. Sultana, R., et al., *Awareness and Lifestyle Practices among Type-II Diabetics Pertaining to the Disease Attended at Outpatient Clinics of Nawabshah, Shaheed Benazirabad.* Journal of Pharmaceutical Research International, 2021. **33**(29A): p. 67-75.
6. Ramzan, B., et al., *Impact of diabetes-related knowledge and medication adherence on quality of life among type 2 diabetes patients in a tertiary health facility in Multan, Pakistan.* Tropical Journal of Pharmaceutical Research, 2022. **21**(4): p. 871-877.
7. Iqbal, Q., et al., *Profile and predictors of health related quality of life among type II diabetes mellitus patients in Quetta city, Pakistan.* Health and Quality of Life Outcomes, 2017. **15**(1): p. 142.
8. Khan, A., et al., *AWARENESS REGARDING DIABETES AND ITS MANAGEMENT AMONGST PATIENTS VISITING TERTIARY CARE HOSPITALS AND ITS IMPACT ON GLYCEMIC CONTROL.* Pakistan Armed Forces Medical Journal, 2021. **71**(5): p. 1815-19.

**Diabetes Treatment Adherence**

1. Khowaja, M.A., et al., *Factors associated with Diabetes Empowerment among patients with type 2 diabetes, at OPD setting, Karachi, Pakistan.* Scientific Reports, 2023. **13**(1): p. 7165.
2. Ramzan, B., et al., *Impact of diabetes-related knowledge and medication adherence on quality of life among type 2 diabetes patients in a tertiary health facility in Multan, Pakistan.* Tropical Journal of Pharmaceutical Research, 2022. **21**(4): p. 871-877
3. Iqbal, Q., et al., *Profile and predictors of health related quality of life among type II diabetes mellitus patients in Quetta city, Pakistan.* Health and Quality of Life Outcomes, 2017. **15**(1): p. 142.
4. Butt, M.D., et al., *An observational multi-center study on type 2 diabetes treatment prescribing pattern and patient adherence to treatment.* Sci Rep, 2023. **13**(1): p. 23037
5. Shams, N., et al., *Geriatric Type 2 diabetes; the risk factors and associations for medication non-adherence.* The Professional Medical Journal, 2020. **27**(02): p. 260-267.
6. Beg, B.M., et al., *EVALUATION OF MEDICATION ADHERENCE AMONG DIABETICS: A CROSS-SECTIONAL STUDY IN LAHORE, PUNJAB, PAKISTAN.* Independent Journal of Allied Health Sciences, 2018. **1**(01): p. 27-32.
7. Nazir, S.U.R., et al., *Disease related knowledge, medication adherence and glycaemic control among patients with type 2 diabetes mellitus in Pakistan.* Primary care diabetes, 2016. **10**(2): p. 136-141.
8. Sarwar, H., et al., *Adherence with Prescribed Medications Among Diabetes Mellitus II Patients.* Int J Pharm Sci Rev Res, 2014. **24**: p. 246-252.

**Diabetes Complications**

1. Basit, A., et al., *Frequency of chronic complications of type 2 diabetes.* J Coll Physicians Surg Pak, 2004. **14**(2): p. 79-83.
2. Zia, A., et al., *Prevalence of type 2 diabetes–associated complications in Pakistan.* International Journal of Diabetes in Developing Countries, 2016. **36**(2): p. 179-188.
3. Uddin, F., B. Ali, and N. Junaid, *Prevalence of diabetic complications in newly diagnosed type 2 diabetes patients in Pakistan: findings from national registry.* Journal of Ayub Medical College Abbottabad, 2019. **30**(4-Sup).
4. Abro, M., et al., *Prevalence of diabetic micro vascular complications at a tertiary care unit of Karachi, Pakistan.* International Journal of Diabetes in Developing Countries, 2019. **39**(2): p. 325-330.
5. Asghar, S., et al., *Metabolic Syndrome in Type 2 Diabetes Mellitus Patients: Prevalence, Risk Factors, and Associated Microvascular Complications.* Cureus, 2023. **15**(5).
6. Sharif, S., et al., *Frequency of depression in patients with type 2 diabetes mellitus and its relationship with glycemic control and diabetic microvascular complications.* Cureus, 2019. **11**(7).
7. Akhtar, M.S., et al., *Prevalence of diabetic complications in an urban district of Jhang (Punjab) Pakistan.* Can J App Sci, 2011. **1**(2): p. 43-9.
8. Wahab, S., et al., *Frequency of retinopathy in newly diagnosed type 2 diabetes patients.* JPMA. The journal of the Pakistan Medical Association, 2008. **58**(10): p. 557.
9. Khan, K.A., et al., *FREQUENCY OF RETINOPATHY IN NEWLY DIAGNOSED PATIENTS OF TYPE 2 DIABETES MELLITUS (DM): Retinopathy in Type 2 DM.* Pakistan Armed Forces Medical Journal, 2015. **65**(1): p. 63-67.
10. Hussain, S., et al., *Risk factors of retinopathy in type 2 diabetes mellitus at a tertiary care hospital, Bahawalpur Pakistan.* Pak J Med Sci, 2013. **29**(2): p. 536-9.
11. TAHIR, M.M., et al., *Frequency of Peripheral Neuropathy in Newly Diagnosed Type 2 Diabetes Presenting in a Tertiary Care Hospital of Lahore.*
12. Shera AS, Jawad F, Maqsood A, Jamal S, Azfar M, Ahmed U. Prevalence of chronic complications and associated factors in type 2 diabetes. J Pak Med Assoc. 2004;54(2):54-9.

**Cost and Financial Burden**

1. Butt, M.D., et al., *Cost of Illness Analysis of Type 2 Diabetes Mellitus: The Findings from a Lower-Middle Income Country.* International Journal of Environmental Research and Public Health, 2022. **19**(19): p. 12611.
2. Khowaja, L.A., A.K. Khuwaja, and P. Cosgrove, *Cost of diabetes care in out-patient clinics of Karachi, Pakistan.* BMC Health Services Research, 2007. **7**(1): p. 189.
3. Datta, B.K., H. Muhammad Jami, and S. Asma, *Assessing the relationship between out-of-pocket spending on blood pressure and diabetes medication and household catastrophic health expenditure: evidence from Pakistan.* International Journal for Equity in Health, 2019. **18**.
4. Hussain, M., et al., *Direct cost of treatment of diabetes mellitus type 2 in Pakistan.* Int J Pharm Pharm Sci, 2014. **6**(11): p. 261-4.
